# Rebranding social distancing to physical distancing: calling for a change in the health promotion vocabulary to enhance clear communication during a pandemic

**DOI:** 10.1177/1757975920986126

**Published:** 2021-01-24

**Authors:** Kristine Sørensen, Orkan Okan, Barbara Kondilis, Diane Levin-Zamir

**Affiliations:** 1 Global Health Literacy Academy, Denmark; 2 University of Education Freiburg, Germany; 3 I-Shou University, Taïwan; 4 Bielefeld University, Germany; 5 Hellenic American College/Hellenic American University, Athens, Greece; 6 Clalit Health Services, Israel; 7 Haifa University, Israel

**Keywords:** pandemic, social distancing, physical distancing, spatial distancing, non-pharmaceutical measures, health literacy

## Abstract

Amidst the COVID-19 outbreak, the term ‘social distancing’ received immense attention in the mainstream and social media and was embraced by governments as a universal precaution to stem the coronavirus pandemic. ‘Social distancing’ belongs technically to a set of non-pharmaceutical infection control actions intended to stop or slow down the spread of a contagious disease. However, several weeks into the outbreak, scholars discussed whether the term was, in fact, misleading and could be counterproductive. To study the arguments, the study design included (1) analysis of the performance of the concepts ‘social distancing’ and ‘physical distancing’ based on Google Trends (15 February–15 June 2020); (2) analysis of the arguments used in media discussions of ‘social distancing versus physical distancing’ in the period 15 March–15 April 2020, derived from a Google search; and (3) a scientific literature review in PubMed. The study was conducted in English. The trend analysis showed the peak and the decrease of the relative popularity of ‘social distancing’ and ‘physical distancing’ during spring 2020. The thematic analysis of Google sources yielded an overview of arguments based on nine themes with two to five sub-themes reflecting on the misleading concept, the historical perspective, the sociological perspective, the public health perspective, alternative proposals regarding the social and the physical dimensions, the distinction of terms, the political choice, and the need for rebranding. Two papers were included in the scientific literature review, which both stressed the need for a change of terminology. In conclusion, the study emphasizes that the choice of terminology matters when life-saving public health messages are designed. It is therefore recommended to rebrand ‘social distancing’ to ‘physical distancing’ to enhance clear communication during the current COVID-19 pandemic in order to prepare for future pandemics.

## Introduction

Amidst the COVID-19 outbreak, the term ‘social distancing’ received tremendous attention in the mainstream and social media and was embraced by governments as a universal precaution to stem the coronavirus pandemic. ‘Social distancing’ belongs technically to a set of non-pharmaceutical infection control actions intended to stop or slow down the spread of a contagious disease. As the COVID-19 outbreak evolved, the act of ‘social distancing’ was deployed as a protective means in countries across the world with varying degrees of intensity, yet often with high costs to individuals and societies (1,2).

While ‘social distancing’ measures date back to at least the 5th century BC, the earliest reference in English can be found in the 1831 translation of Louis Antoine Fauvelet de Bourrienne’s memoirs of his friendship with Napoleon. Later the term was used as a euphuism for social class and applied to the Social Distance Scale created by sociologist Emory Bogardus in 1919 measuring prejudice by asking participants to describe how comfortable they feel interacting with people of another race (3).

In 1963 Edward Hall, a cultural anthropologist, coined the term *proxemics* to define studies about ‘social distancing’ in everyday life. His concern was that closer distances between two persons may increase visual, tactile, auditory, or olfactory stimulation to the point that some people may feel intruded upon and react negatively, and he proposed four main zones of space between individuals:

- Intimate distance (less than half a metre), such as in giving or receiving a hug.
- Personal distance (about 1 metre), usually reserved for family or good friends.
- Social distance (2 to 3 metres), when meeting strangers.
- Public distance (more than 5 metres), such as in public presentations.

‘Social distancing’ was used to prevent disease when it was applied during the HIV and SARS epidemics (4). As of today, the rationale for deploying the distancing measure in public is that COVID-19 spreads through means such as touching, coughing and sneezing. Hence, if people are spaced far enough apart, they will be out of reach of being exposed to the coronavirus (5). Any break in a chain of contacts breaks the disease transmission along that chain, which is why distancing measures are highly recommended during an epidemic such as COVID-19 (6).

Interestingly, several weeks into the outbreak, scholars discussed whether the term ‘social distancing’ was, in fact, misleading and could be counterproductive (7). It was stressed that it was about ‘distant socialization’ rather than ‘social distancing’ (3) and that the ‘efforts taken to slow the spread of the coronavirus should encourage strengthening social ties while maintaining physical distancing (7)’. The Secretary-General of the World Health Organization, Dr Tedros Adhanom Ghebreyesus, quickly adapted the new term ‘physical distancing’ in his announcements (1). To gain clarity, the aim of this study was, therefore, to assess the arguments associated with the use of the terms ‘social distancing’ and ‘physical distancing’ and apply a health literacy lens to the discussion of the outcomes to guide their use in the realm of public health and health promotion during the current COVID-19 pandemic and in the future.

## Methods

The discussion of ‘social distancing versus physical distancing’ was reviewed based on a two-tier strategy – peer-reviewed articles as well as grey literature derived from Internet searches – to reflect the real-life discussion outside the academic field. The study was conducted in English. Firstly, a trend analysis was conducted in Google Trends to explore the performance of the terms worldwide in the period of 15 February–15 June 2020. Google Trends provides access to a largely unfiltered sample of actual search requests made to Google. It is anonymized, categorized and aggregated to display the interest in a particular topic from around the globe or down-to-city-level geography. Google Trends shows the ‘relative popularity’ of a search query to make comparisons easier. Each data point is divided by the total searches of the geography and time range it represents, to compare relative popularity. The resulting numbers are scaled on a range from 0 to 100 based on the topics’ proportion to all searches (8). Secondly, an analysis of sources derived from Google was conducted based on the search terms ‘social distancing vs physical distancing’, ‘social distancing versus physical distancing’ and ‘social distancing and physical distancing’ covering the period of 15 March–15 April 2020. This period included the peak according to the Google Trends analysis. The sources were retrieved and analysed thematically to gain insights into the arguments used. Thirdly, a literature search was conducted in April and updated in July 2020 in PubMed using the search words ‘social distancing’ and ‘physical distancing’, respectively, in the title/abstract. The abstracts were scanned for relevance to see if they reflected the discussion regarding the change of terminology. If included, full texts were retrieved for the selected articles and analysed. The findings were analysed to make a final synthesis of the results.

## Results

### Google trend analysis

**Figure 1.**
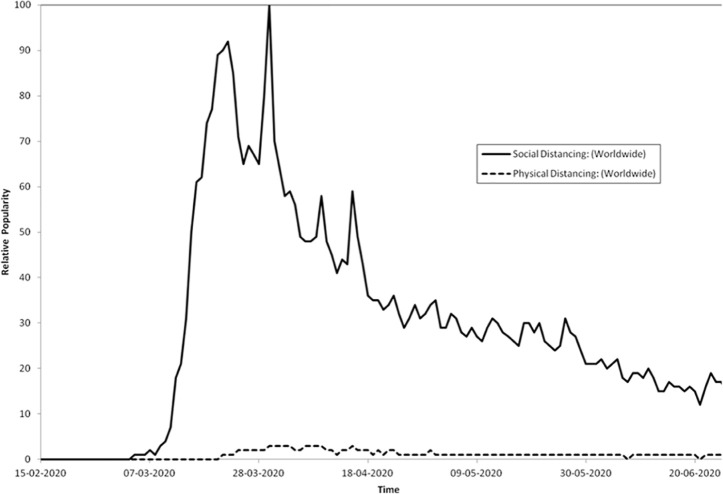
Google trend analysis of the terms social distancing and physical distancing.

The trend analysis revealed increased activity of the term ‘social distancing’ at the beginning of March with peaks in mid-March and end of March 2020. Shortly after, at the end of March and beginning of April, ‘physical distancing’ appeared as a search term; however, it never reached the same attention as ‘social distance’. The proportions were the same when the search terms were substituted with ‘social distance’ and ‘physical distance’. From April to June 2020, the relative popularity decreased (Figure 1).

### Analysis of Google sources

**Figure 2.**
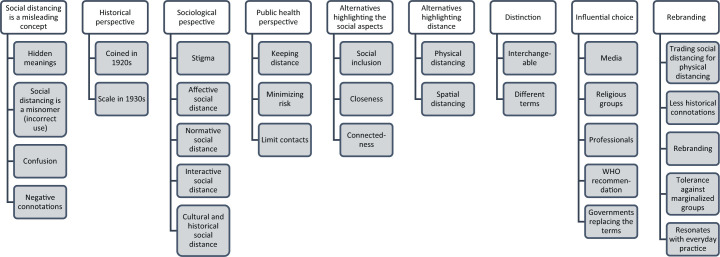
Thematic analysis of arguments concerning social distancing versus physical distancing.

The Google search yielded *n* = 31 results for ‘social distancing vs physical distancing’; *n* = 5 for ‘social distancing versus physical distancing’ and *n* = 57 for ‘social distancing AND physical distancing’, making a total of 88 results when duplicates were removed. These sources were reviewed for relevance and *n *= 33 were included in the study. The entities in the sources represented media, individuals, faith-based groups, health professionals, health organizations, schools, and governments. An inductive thematic analysis of sources resulted in nine themes with 2–5 sub-themes describing the arguments put forward by the stakeholders discussing the use of ‘social distancing’ versus ‘physical distancing’. The themes are outlined in Figure 2 and described in detail below grounded in the content of the sources.

#### 1. Misleading concept

A prominent theme in the study highlighted that the concept of ‘social distancing’ was misleading and can be misunderstood. It has hidden meanings based on the various ways that it can be interpreted. In several cases, it was characterized as a ‘misnomer’, meaning a term that is used incorrectly. The term was also described as a term with negative connotations due to its association with ‘social isolation’. Notably, it was mentioned that the way in which the concept was used in the coronavirus pandemic could create confusion and unclarity.

But social distancing is a bit of misnomer it should actually be called physical distancing (#22- media).

#### 2. Historical perspective

A minor theme represented how one website referred to the historical perspective on the use of ‘social distancing’.

It was coined in the 1920s by sociologist Robert Park. In the 1930s the Bogardus social distance scale was developed to measure people’s desire to keep a distance from individuals in socially devalued groups (#25-media).

#### 3. Public health perspective

The analysis revealed that in public health contexts, ‘social distancing’ generally referred to various measures that reduce close contact (increase distance) between large groups of people (hence social). Yet, in most of the included links it was followed by an explanation like this:

However, to stay healthy, this is about physical separation, not social segregation and it’s an important distinction (#1-health professional).

When the epidemiologists say ‘social distancing,’ they don’t mean don’t talk to friends and family. In contrast, they highly encourage it. It is a better idea than ever to call your parents or grandparents. FaceTime your friends. Make sure that you are talking to people and staying plugged in. Therefore, we shouldn’t call it ‘social distancing.’ Perhaps ‘physical distancing’ is a better term (#33-professional).

Yet, the term ‘social distancing’ was widely applied in a public health context and recommended, for example, by the U.S. Centers for Disease Control and Prevention (CDC) as an effective measure to help curb the spread of respiratory illnesses such as coronavirus. A CDC link stated:

According to the CDC, social distancing involves ‘remaining out of congregate settings, avoiding mass gatherings, and maintaining distance (approximately 6 feet or 2 meters) from others when possible.’ And congregate settings include ‘crowded public places where close contact with others may occur, such as shopping centers, movie theatres, stadiums’ (#30-professional).

#### 4. Sociological perspective

A unique theme in the study, however, also revealed that ‘social distancing’ could be confused with the concept of social distance in sociology, which means ‘the extent to which individuals or groups are removed from or excluded from participating in one another’s lives’. It was highlighted that ‘social distancing’ could be viewed as having various dimensions (#25-media). Specifically, it is associated with the affective distance, which refers to how much sympathy the members of a group feel for another group. It was mentioned that Emory Bogardus, the creator of the Bogardus Social Distance Scale, based the instrument on the subjective–affective concept of social distance: the feeling reactions of persons toward other persons and groups of people in terms of attitude and prejudice (#25-media). Besides, it was stated that a second approach views social distance as a normative category which refers to the widely accepted and often consciously expressed norms about who should be considered as an ‘insider’ and who an ‘outsider/foreigner’, hence specifying the distinctions between ‘us’ and ‘them’. These approaches are associated with sociologists such as Georg Simmel, Emile Durkheim and, to some extent, Robert Park. Furthermore, interactive social distance refers to the frequency and intensity of interactions between two groups, claiming that the more the members of the two groups interact, the closer they are socially. This aspect is similar to sociological network theory, where the frequency of interaction between two parties is used as a measure of the strength of the social ties between the parties. Lastly, cultural, and habitual distance proposed by Bourdieu is influenced by the ‘capital’ that people possess (#25-media).

#### 5. Alternatives highlighting the social aspects

A prominent theme stressed the importance of recognizing the signs of solidarity and the social aspect of ‘social distancing’. Several options were suggested, such as ‘social inclusion’, ‘social closeness’, and ‘connectedness’. It was highlighted that:

At a time of crisis and fear, we need the opposite of social distancing. We need a sense that we are in this together, that people are not facing this alone. We need social inclusion, not distance (#1-health professional).

We want to erode social distance and have closeness (#25-media).

We need that sense of closeness with others, those conversations that make us laugh, and those connections of warmth and compassion. It’s not Social Distancing. It is Physical Distancing we are asked to do (#9-individual).

#### 6. Alternatives highlighting distance in various forms

The study included several suggestions to substitute for the concept of ‘social distancing’ in order to not create confusion, namely ‘physical distancing’, and ‘spatial distancing’.

Words matter and other terms that have less historic connotations can be used. Perhaps a good place to start is to rebrand the current ‘social distancing’ to the more accurate ‘spatial distancing’ or ‘physical distancing’. (#25-media).

#### 7. Distinction

The study revealed that some contributors to the discussion made a clear distinction between ‘social distancing’ and ‘spatial distancing’ or ‘physical distancing’. In contrast, others used ‘social distancing’ and ‘physical distancing’ interchangeably.

Social distancing, physical distancing, is there a difference? These two terms are in use everywhere around you. You may be confused, but the answer is simple. The two terms are fully interchangeable (#19-NGO).

It’s so important that we change in our mindset and make a distinction between social distancing and physical distancing. Physical distancing right now is of the utmost importance. There is an increasing imperative right now to be physically apart from one another so that we don’t continue the spread of COVID-19. But that does not mean that we need to practice social distancing (#23-media).

#### 8. Choice of authorities

A theme highlighted in the sources was a call for a change of vocabulary, in the choice made by public authorities. Significant stakeholders such as the World Health Organization (WHO), governments, the media, religious groups and health professionals announced a change transitioning from ‘social distancing’ to ‘physical distancing’. Health professionals increasingly encouraged the use of the term ‘physical distancing’ as a more precise alternative to ‘social distancing’ by arguing that ‘physical distancing’ underscores the importance of maintaining the physical distance between people to help stop the spread of the coronavirus. The term additionally emphasizes that people should still spend time with friends and family using digital technology and social media when they are physically separated. The WHO epidemiologist Dr Maria Van Kerkhove highlighted that the WHO is adopting the use of ‘physical distancing’ instead of ‘social distancing’:

Keeping a physical distance … that’s absolutely essential, but it doesn’t mean that socially we have to disconnect from our loved ones, from our family … We say ‘social distancing’; we are changing to say ‘physical distance’, and that’s on purpose because we want people to still remain connected … Because your mental health going through this is just as important as your physical health. (#3-magazine).

The study also revealed that several governments had taken steps to replace ‘social distancing’ with ‘physical distancing’:

Social distancing is one of the steps to prevent and control Coronavirus infection by encouraging healthy people to limit visits to crowded places and direct contact with others. Now, the term social distancing has been replaced by physical distancing by the government (#24-business).

#### 9. A call for rebranding

Lastly, a common theme concerned the need for rebranding the concepts and trading ‘social distancing’ for ‘physical distancing’. The reasons included that ‘physical distancing’ had less historical and negative connotations, and facilitated tolerance against a marginalized group, rather than signalling or inducing social isolation and exclusion. Furthermore, it was highlighted that ‘physical distancing’ resonated with everyday practice and can be understood in clear terms rather than having to be explained in plain language.

Words matter and other terms that have less historic connotations can be used. Perhaps a good place to start is to rebrand the current ‘social distancing’ to the more accurate ‘spatial distancing’ or ‘physical distancing’ (#25-media).

### Scientific literature review

The assessment of the use of the terms ‘social distancing’ and ‘physical distancing’ in PubMed revealed that ‘social distancing’ (*n* = 1092) was most commonly used in comparison to ‘physical distancing’ (*n* = 154) in a search based on title/abstract (last updated 10 July 2020). The search ‘social distancing AND physical distancing’ in title/abstract yielded 20 references which were reviewed in terms of relevance, resulting in the inclusion of two papers by Wasserman *et al*. (9) and Bergman *et al*. (10).

Bergman *et al*. (10) explored the importance of encounters in the health system, recognizing the great potential to create a positive change in a health care system that currently feels fragmented and depersonalized to both patients and health care clinicians through the many ways of investing and supporting relationships. They believe that the current COVID-19 pandemic offers unique opportunities to use remote communication to develop healing human relationships and enhance social connectedness.

Human connectedness — love — is more contagious than coronavirus. What we need now is not social distancing, but physical distancing with social connectedness (10).

Wasserman *et al*. (9) stated that policymakers, media, governments, and the general public should be encouraged to use the more neutral term of ‘physical distancing’ rather than ‘social distancing’ during the COVID-19 pandemic, based on the negative connotations of this term. As COVID-19 has plagued the world, the term ‘social distancing’ has been widely used to encourage the general population to physically distance themselves from others to reduce the spread of the virus. They argue that this term can have unintended detrimental effects, as it can evoke negative feelings of being ignored, unwelcome, left alone with one’s fears, and even excluded from society.

Essentially, both papers argue for a change. Bergman *et al*. focus on the importance of social connectedness rather than social distance and Wasserman *et al*. encourage substituting ‘social distancing’ with ‘physical distancing’ to adopt a more neutral term instead of the negatively loaded concept of ‘social distancing’.

### Synthesis

This research revealed that the use of ‘social distancing’ in a public health context could be misunderstood due to the historical and sociological applications of the concepts. In addition, the study showed that the concepts of ‘social distancing’ and ‘physical distancing’ are either applied interchangeably or regarded as distinct concepts. Alternatives were suggested which focused on social dimensions such as connectedness and closeness. Other proposals focused on the *distancing* aspects like the terms ‘physical distancing’ and ‘spatial distancing’. By the end of March 2020, significant stakeholders such as the WHO and governments called for making a change by phasing out the use of ‘social distancing’ and replacing it with ‘physical distancing’. A rebranding of the terms was encouraged to facilitate clarity and mobilize a more widespread change. The arguments included avoiding stigma, enhancing disease prevention and basing the word choice on terminology that is less historically embedded and neutral rather than negatively connoted. However, the trend analysis also highlighted that the popularity of the term ‘social distancing’ has been decreasing since April 2020 and that the term ‘physical distancing’ was not at any time level with the use of the term ‘social distancing’.

## Discussion

Initially, the concept of ‘social distancing’ was applied from the end of February 2020 as a non-pharmaceutical instrument in a public health context to conquer the coronavirus pandemic. However, a vigorous discussion was initiated in media and social media, arguing for a change of terminology. Hence, this study was made to provide deeper insights into the conceptual underpinning of distancing measures and why a call was made to change the vocabulary. The study advised rebranding the concept of ‘social distancing’ to ‘physical distancing’ for various reasons. The results resonate with the opinion expressed by experts in other fields, such as health promotion (11) and sociology (12). The arguments against a change indicate that ‘social distancing’ has already taken root. It is argued that people do understand what it is and that they adopt it as individuals alongside organizations applying this protective action. To maintain consistent messages from trusted sources, the terminology should not be altered. Confusion could be dangerous because it is a matter of life and death when it comes to the COVID-19. In contrast, some advocate for changing from ‘social’ to ‘spatial’ to emphasize that the focus of action is on taking physical distance, rather than distancing oneself socially and emotionally from others, which is important especially from a health promotion perspective (11,12).

The main arguments for the change concern the embedded paradox in the term ‘social distancing’. In the act of ‘social distancing’, people are collaborative and social by mutually agreeing to stay apart from each other. Yet, the term ‘social distancing’ might also imply a risk that ‘social distancing’ could exacerbate already existing social division in society. Although it may seem clear that ‘social distancing’ is a physical, not a social, requirement, some people might think it is about being solitary in their homes. More importantly, social connections are necessary not just to overcome the pandemic, but for rebuilding and recovering. Collaborative, mutually supportive communities are the ones that are most successful at sustainably recovering from large disasters and long-term crisis (13). Yet, the pandemic circumstances have revealed how difficult it is to avoid social isolation when applying the distance measures to broader society. The elderly, in particular, for whom distancing is detrimental, have suffered due to the minimization of social contacts (14). Indeed, the paradox prevailing during the epidemic is that while ‘social distancing’ is required to contain the spread of the coronavirus, it may also contribute to poor health in the long run due to lack of exercise and mental health issues (15). Being mentally healthy is more vital than ever during an epidemic (1). Therefore, replacing the term ‘social distancing’ with ‘physical distancing’ is not just a semantic distinction.

The present study has some limitations. The narrow focus on ‘social distancing versus physical distancing’ may have left out relevant material which could have brought more nuances to the list of arguments for and against a change. Likewise, more proposals for new terms could have been generated, such as, for example, ‘geographical distance’. Hence, more research is warranted to guide the terminology choice in the future.

When many governments have implemented varying forms of lockdowns (on a spectrum from ‘light’ to ‘strict’ lockdowns to stay-home-orders to curfews to ‘hard’ quarantines) or have insisted on distancing measures as part of their national response to the COVID-19 outbreak, the populations must interpret and understand what it means and why it matters. Being able to comprehend public health recommendations and use the specific knowledge to inform action and behaviour is key for curbing the pandemic. Communicating risk by using effective terminology and use of appropriate language, images and/or statistics in health material is critical for health behaviour change (16). It is why health literacy is so crucial in the time of the COVID-19 epidemic globally (17). Health literacy is defined as people’s knowledge, motivation and competencies to access, understand, appraise and apply information and services to manage health (18). In turn, this is related to the complexity of the information and services provided by authorities (19,20). Yet, health literacy is a public health challenge that should not be neglected by decision-makers and professionals working with health communication and service design, especially not in a time of crisis (21). Conversely, clear and concise information is a necessity in dealing with the complexity of the COVID-19 outbreak (4,22,23).

While the study was conducted in spring 2020 at the beginning of the pandemic, the way the terms are adopted is becoming clearer with time. Over the summer, practice indicated that in several languages the terms ‘social distancing’ is difficult to translate and barely or not used with regards to disease prevention. Moreover, while authorities might have eventually adopted both terms, it seems that they use them interchangeably, as seen in the examples from the Centers of Disease Control in the USA and National Health Services from the UK. Yet, the fact that ‘physical distancing’ is mentioned as part of the official communication indicates that it is at least accepted as a valid prevention measure against COVID-19.

Social distancing, also called ‘physical distancing’, means keeping a safe space between yourself and other people who are not from your household.

Physical distancing measures are things you should do to reduce how often you interact with others outside your household. This will stop coronavirus (COVID-19) spreading (24).

Social distancing measures (also known as physical distancing) are steps you can take to reduce very close or physical contact between yourself and other people. This will help reduce the transmission of coronavirus (COVID-19) (25).

In conclusion, this paper reflected on the use of terminology about distancing measures to guide a more consistent communicative approach in the future. Introducing a term that is not well known by many is always risky. Choosing a term that is known and applied in one scientific area (sociology) with a different connotation in another (public health) adds to the confusion. When the construct ‘social distance’ is verbalized to a new construct ‘social distancing’, it might add to the confusion even more as it can be misinterpreted as ‘social isolation’. The study emphasized that the choice of terminology matters when life-saving public health messages are designed. It is therefore recommended to rebrand ‘social distancing’ to ‘physical distancing’ to enhance clear communication during the current COVID-19 pandemic in order to better prepare for future pandemics. Yet, in practice, while ‘physical distancing’ is being adopted by authorities, it seems to be applied interchangeably with ‘social distancing’. More research is warranted to implement distancing measures in a health-literate manner.

## References

[1] Hart M. WHO Changes ‘Social Distancing’ to ‘Physical Distancing’ [Internet]. Nerdist; 2020 [cited 2020 Apr 6]. Available from: https://nerdist.com/article/social-distancing-changed-physical-distancing/

[2] West R Michie S Rubin GJ Amlôt R. Applying principles of behaviour change to reduce SARS-CoV-2 transmission. Nat Hum Behav. 2020; 4: 451–459. 32377018 10.1038/s41562-020-0887-9

[3] Sørensen K. “Let us carry out #socialdistancing with a focus on #distantsocialization keeping us closer rather than bringing us apart #mentalhealthliteracy #healthliteracy #distantparticipation” [Internet]. 2020 [cited 2020 Apr 6]. Available from: https://twitter.com/k_srensen/status/1238188317637828615

[4] Okan O Sørensen K Messer M. COVID-19: a guide to good practice on keeping people well informed; [Internet]. 2020 [cited 2020 Jul 13]. Available from: https://theconversation.com/covid-19-a-guide-to-good-practice-on-keeping-people-well-informed-134046

[5] World Health Organization. Coronavirus Disease (COVID-19) Advice for the Public [Internet]. World Health Organization; 2020 [cited 2020 Apr 6]. Available from: https://www.who.int/emergencies/diseases/novel-coronavirus-2019/advice-for-public

[6] Ladra. Epidemiologist Explains Why Social Distancing is #1 Weapon vs COVID19 –: Political Cortadito [Internet]. 2020 [cited 2020 Apr 6]. Available from: http://www.politicalcortadito.com/2020/03/24/epidemiologist-explains-why-social-distancing-is-1-weapon-vs-covid19/

[7] Gale R. Is ‘Social Distancing’ The Wrong Term? Expert Prefers ‘Physical Distancing,’ and The WHO Agrees [Internet]. *The Washington Post*; 2020 [cited 2020 Apr 6]. Available from: https://www.washingtonpost.com/lifestyle/wellness/social-distancing-coronavirus-physical-distancing/2020/03/25/a4d4b8bc-6ecf-11ea-aa80-c2470c6b2034_story.html

[8] FAQ about Google Trends Data - Trends Help [Internet]. 2020 [cited 2020 Jul 13]. Available from: https://support.google.com/trends/answer/4365533?hl=en

[9] Wasserman D van der Gaag R Wise J. The term “physical distancing” is recommended rather than “social distancing” during the COVID-19 pandemic for reducing feelings of rejection among people with mental health problems. Eur Psychiatry. 2020; 63: e52. 10.1192/j.eurpsy.2020.60 PMC7287304 32475365

[10] Bergman D Bethell C Gombojav N Hassink S Stange KC. Physical distancing with social connectedness. Ann Fam Med. 2020; 18: 272–277. 32393566 10.1370/afm.2538 PMC7213990

[11] van den Broucke S. Why health promotion matters to the COVID-19 pandemic, and vice versa. Health Promot Int. 2020; 35:181–186. Available from: https://academic.oup.com/heapro/article/35/2/181/5820891. 32297931 10.1093/heapro/daaa042 PMC7184433

[12] Abel T McQueen D. The COVID-19 pandemic calls for spatial distancing and social closeness: not for social distancing! Int J Public Health 2020. 10.1007/s00038-020-01366-7 PMC7111296 32239256

[13] Wilder-Smith A Freedman DO. Isolation, quarantine, social distancing and community containment: pivotal role for old-style public health measures in the novel coronavirus (2019-nCoV) outbreak. J Travel Med 2020; 27. 10.1093/jtm/taaa020 PMC7107565 32052841

[14] Ramage-Morin PL Polsky JY. Health-related concerns and precautions during the COVID-19 pandemic: a comparison of Canadians with and without underlying health conditions. Health Rep. 2020; 31: 3–8. 10.25318/82-003-x202000500001-eng 32644765

[15] Miller KE. Let’s Aim for physical rather than social distancing: isolation can be toxic. Let’s increase physical distance but stay connected [Internet]. *Psychology Today*; 2020 [cited 2020 Apr 6]. Available from: https://www.psychologytoday.com/us/blog/the-refugee-experience/202003/lets-aim-physical-rather-social-distancing

[16] Zarcadoolas C Pleasant A Greer DS. Understanding health literacy: an expanded model. Health Promot Int. 2005; 20: 195–203. 15788526 10.1093/heapro/dah609

[17] Košir U Sørensen K. COVID-19: the key to flattening the curve is health literacy. Perspect Public Health. 2020; 1757913920936717. 10.1177/1757913920936717 32650704

[18] Sørensen K van den Broucke S Fullam J Doyle G Pelikan J Slonska Z, et al. Health literacy and public health: a systematic review and integration of definitions and models. BMC Public Health. 2012; 12: 80. 22276600 10.1186/1471-2458-12-80 PMC3292515

[19] Parker R Ratzan SC. Health literacy: a second decade of distinction for Americans. J Health Commun. 2010; 15(Suppl 2): 20–33. 20845190 10.1080/10810730.2010.501094

[20] Sørensen K Trezona A Levin-Zamir D Kosir U Nutbeam D. Transforming health systems and societies by investing in health literacy policy and strategy. Public Health Panorama. 2019; 5: 259–263.

[21] Sørensen K Pelikan JM Röthlin F Ganahl K Slonska Z Doyle G, et al. Health literacy in Europe: comparative results of the European health literacy survey (HLS-EU). Eur J Public Health. 2015; 25: 1053–1058. 25843827 10.1093/eurpub/ckv043 PMC4668324

[22] International Health Literacy Association. IHLA Statement for the WHO General Assembly [Internet]. 2020 [cited 2020 Jul 13]. Available from: http://www.i-hla.org/ihla-statement-for-the-who-general-assembly/

[23] Chu DK Akl EA Duda S Solo K Yaacoub S Schünemann HJ, et al. Physical distancing, face masks, and eye protection to prevent person-to-person transmission of SARS-CoV-2 and COVID-19: a systematic review and meta-analysis. Lancet 2020; 395: 1973–1987. 32497510 10.1016/S0140-6736(20)31142-9 PMC7263814

[24] CDC. Social Distancing [Internet]. 2020 [cited 2020 Jul 13]. Available from: https://www.cdc.gov/coronavirus/2019-ncov/prevent-getting-sick/social-distancing.html

[25] NHS Wales [Internet]. 2020 [cited 2020 Jul 13]. Available from: https://gov.wales/coronavirus-social-distancing-guidance#:~:text=Keep%20Wales%20safe%3A,your%20extended%20household%2C%20stay%20outdoors

